# Enhanced Antibacterial Effect on Zirconia Implant Abutment by Silver Linear-Beam Ion Implantation

**DOI:** 10.3390/jfb14010046

**Published:** 2023-01-13

**Authors:** Yang Yang, Mingyue Liu, Zhen Yang, Wei-Shao Lin, Li Chen, Jianguo Tan

**Affiliations:** 1 Department of Prosthodontics, Peking University School, Hospital of Stomatology, Beijing 100081, China; 2 National Center of Stomatology, National Clinical Research Center for Oral Diseases, National Engineering Research Center of Oral Biomaterials and Digital Medical Devices, Beijing Key Laboratory of Digital Stomatology, Research Center of Engineering and Technology for Computerized Dentistry Ministry of Health, NMPA Key Laboratory for Dental Materials, No. 22, Zhongguancun South Avenue, Haidian District, Beijing 100081, China; 3 First Clinical Division, Peking University School, Hospital of Stomatology, Beijing 100081, China; 4 Department of Prosthodontics, Indiana University School of Dentistry, Indianapolis, IN 46202, USA

**Keywords:** zirconia, silver, ion implantation, peri-implant lesions, implant interface

## Abstract

Peri-implant lesions, such as peri-implant mucositis and peri-implantitis, are bacterial-derived diseases that happen around dental implants, compromising the long-term stability and esthetics of implant restoration. Here, we report a surface-modification method on zirconia implant abutment using silver linear-beam ion implantation to reduce the bacterial growth around the implant site, thereby decreasing the prevalence of peri-implant lesions. The surface characteristics of zirconia after ion implantation was evaluated using energy dispersive spectroscopy, X-ray photoelectron spectroscopy, and a contact-angle device. The antibacterial properties of implanted zirconia were evaluated using *Streptococcus mutans* and *Porphyromonas gingivalis*. The biocompatibility of the material surface was evaluated using human gingival fibroblasts. Our study shows that the zirconia surface was successfully modified with silver nanoparticles by using the ion-implantation method. The surface modification remained stable, and the silver-ion elution was below 1 ppm after one-month of storage. The modified surface can effectively eliminate bacterial growth, while the normal gingiva’s cell growth is not interfered with. The results of the study demonstrate that a silver-ion-implanted zirconia surface possesses good antibacterial properties and good biocompatibility. The surface modification using silver-ion implantation is a promising method for future usage.

## 1. Introduction

Dental implantation is now widely applied as a reliable method to restore missing human teeth. Successful implants rely not only on solid integration with the surrounding bone tissues (osseointegration), but also on soft-tissue integration. A robust soft-tissue integration can act as an intact seal, preventing oral bacterial invasion, as well as providing good esthetics [1]. When the soft-tissue seal is interrupted or destructed by oral bacteria, the implant stability is destroyed. There are two types of peri-implant lesions that happen frequently around implant-surrounding tissues: peri-implant mucositis and peri-implantitis [2]. Peri-implant mucositis is a localized infection in the surrounding soft tissues, while peri-implantitis endangers the integrated bones underneath. The prevalence of these two peri-implant lesions is growing at a high speed. A study in 2019 reported that 34% of patients experienced peri-implantitis within 2 years of implant placement [3]. The prevalence of peri-implant mucositis is even higher, since it is believed that most cases of peri-implantitis are derived from peri-implant mucositis [4]. Oral bacteria are one of the major causes of peri-implant lesions, since more and more studies confirm that biofilm accumulation leads to the occurrence of peri-implant mucositis and eventually evolves into peri-implantitis [5]. *Porphyromonas gingivalis*, an anaerobic bacterium and an opportunistic pathogen, has been identified as a pathogen around peri-implant. It has been recognized as a late colonizer that adheres to the extracellular matrix formed by early colonizers such as *Streptococcus mutans* [6]. These bacteria synergistically contribute to the emergence of peri-implant lesions [7,8].

The management of peri-implant lesions is difficult and costly. The commonly used non-surgical approaches include scaling, sand-blasting, laser treatment, and localized/general antibiotic administration; the surgical approaches include periodontal flap surgery, guided tissue regeneration, and other regeneration therapies [9]. If these interventions fail, the implant might eventually be removed. An alternative perspective of peri-implant lesions management is to prevent them from emerging [10]. Since peri-implant lesions are recognized as bacterial-derived diseases, plaque control around the implant site can effectively reduce its prevalence. On the other hand, the managing of peri-implant mucositis can reduce the prevalence of peri-implantitis [11], as peri-implant mucositis is recognized as a precursor of peri-implantitis. Over the years, the surface antibacterial-modification method of implant-abutment materials has been an interesting topic to prevent peri-implant lesions. Numerous studies have proved that the surface modification on the implant-abutment surface has the potential to reduce bacterial load around the implant site, thereby facilitating the formation of soft-tissue seals and reducing the emergence of peri-implant lesions [12]. Different surface-modification methods of titanium abutments and implants have been investigated by researchers; the use of either organic or inorganic antibacterial agents on titanium surfaces showed effective bacterial-inhibition efficacy [13,14]. Compared with the organic antibacterial agents, the inorganic antibacterial agents show better stability and greater antibacterial ability. Inorganic antibacterial agents such as silver, copper, zinc, and their oxides possess a broader antibacterial spectrum [15]. Among all inorganic antibacterial agents, silver has been extensively utilized due to its excellent antimicrobial potency. Although widely used in wound-dressing materials, the cell toxicity of silver remains under debate nowadays.

Recent studies revealed that silver poses little biocidal threat to normal cells when at a nano-sized state [16,17]. The silver nanoparticles (AgNPs) cause bacterial death mainly by direct contact with bacterial cell walls [18]. The electrolyte of AgNPs into silver ions can also inhibit bacterial growth without interfering with normal living cells [19]. There is a study that prepared AgNPs with positive or negative charges, and it proved useful in the antibacterial aspect [20]. Among the many ways to fabricate AgNPs, ion-implantation technology showed effectiveness to incorporate AgNPs into solid surfaces. Ion-implantation technology has been widely used in semiconductor device fabrication, metal finishing, as well as materials-science research. There are mainly two types of ion implantation: linear-beam ion implantation and plasma-induced ion implantation [21]. Compared with plasma-induced ion implantation, linear-beam ion implantation can allow to perform an accurate ion implantation through a linear beam to a single point or a flat surface. The size of the implanted nanoparticle is well-controlled through implantation voltage and time [22]. Studies have reported successful silver-nanoparticle implantations on titanium implant surfaces [23,24]. The implanted AgNPs on the implant surface enhanced its anti-microbial efficacy and had little toxicity to normal human cells. More studies have showed that silver-ion implantation is a viable way to modify an object’s surface without changing its original nature [25,26,27,28].

With the development of dental materials, zirconia has gradually become a proper substitute for titanium in implant dentistry. With its ivory color, zirconia implant abutments are believed to be more advantageous than titanium abutments, especially in the esthetic zone [29]. Meanwhile, the zirconia surface attracts less bacteria due to its low surface free energy [30]. However, peri-implant lesions still happen around zirconia implants or abutments [31,32], suggesting that plaque control is still necessary on the zirconia surface. Numerous studies have reported that different methods of surface modification on zirconia surfaces can effectively enhance their antimicrobial properties with good biocompatibilities [33,34,35]. To date, no investigations have laid grounds on silver-ion implantation on zirconia to eliminate bacterial growth, thereby preventing peri-implant lesions. Therefore, this study is designed to explore the method of silver-ion implantation on zirconia surfaces. After the silver-ion implantation, the topography change was evaluated by performing the EDS and XPS analyses and a water-contact-angle device. The antibacterial efficacy was evaluated using *Streptococcus mutans* and *Porphyromonas gingivalis*, and the biocompatibility was evaluated using human gingival fibroblasts. This study provides a viable surface-modification method on zirconia abutments to prevent peri-implant lesions.

## 2. Materials and Methods

### 2.1. Specimen Preparation

Twenty-four yttrium-stabilized zirconia disks (Wieland, Bamberg, Germany) with 15 mm diameter and 2 mm thickness were designed and milled by computer-aided design and computer-aided manufacturing (CAD/CAM) process. An 800-grit SiC abrasive paper was used to polish the disks until a unified roughness height of 0.1 μm was achieved. Then, the zirconia disks were ultrasonically cleaned using absolute ethanol and deionized water, each for 20 min. They were naturally dried and stored at room temperature before silver-ion implantation.

Prior to silver-ion implantation, the zirconia disks were randomly divided into four groups, with six specimens in each group. One group was left untreated and was used as the negative control. The other three groups were implanted using ion bombardment at 30 keV and a nominal dose of 1 × 10^14^ ions/cm^2^, 1 × 10^15^ ions/cm^2^, and 1 × 10^16^ ions/cm^2^, respectively. The implantation energy and nominal dose were determined by preliminary experiments. After ion implantation, the specimens were cooled down to room temperature (25 °C). All specimens were stored at room temperature before use. Before various tests, the disks were sterilized with 75% ethanol for 40 min and washed 3 times using 0.1 M PBS buffer (Solarbio, Beijing, China).

### 2.2. Surface Characteristics

#### 2.2.1. Surface Chemical Composition

The energy dispersive spectroscopy analysis (EDS) was performed to determine surface element composition. The X-ray photoelectron spectroscopy (XPS) (ESCALAB 250; ThermoFisher Scientific, Waltham, MA, USA) examination was performed to further determine the surface chemical composition. In all XPS tests, the survey spectra within the range of 0–600 eV were collected and calibrated using C1s peak at 284.6 eV.

#### 2.2.2. Surface Wettability

The surface wettability was evaluated by measuring the contact angle of 1 μL deionized water droplet using a surface-wettability survey device (OCA15Pro, Dataphysics, Filderstadt, Germany). For each sample, five locations were randomly chosen for the measurement.

### 2.3. Silver-Ion-Elution Test

The zirconia disks with different silver doping were soaked in 10 mL deionized water at 37 °C for 1 day up to 1 month. After the incubation period, the leaching liquid was collected and subjected to inductively coupled plasma mass spectrometry (ICP-MS, iCAP Qc, ThermoFisher Scientific, Waltham, MA, USA). Silver-ion concentration in the liquid was recorded and recognized as a silver release from zirconia disks.

### 2.4. Ethics Statement

This study was approved by the Institutional Review Board of the Peking University School of Stomatology (PKUSSIRB-201943034). The unstimulated human saliva was collected from 5 healthy volunteers, and human gingival fibroblasts (HGFs) were grown from the biopsies obtained from another 10 healthy periodontal human volunteers during periodontal surgery. All participants signed the written informed consent before the procedure.

### 2.5. Bacterial Response

#### 2.5.1. Saliva Coating of Zirconia Disks

Saliva was collected as previously described [36]. Briefly, the whole saliva from healthy donors was collected by unstimulated method and stored on ice. To clarify the saliva samples, they were centrifuged at 3000× *g* for 20 min at 4 °C. After clarification, distilled water was used to dilute the supernatant with a 3:1 ratio *v*/*v*. The 25% saliva was then flited with 0.22 μm PES membrane (Millex GP, Millpore, MA, USA) and stored at −80 °C until use. Prior to the usage, the saliva was immediately thawed at 37 °C and centrifuged again at 1430× *g* for 5 min. All zirconia disks, including the untreated zirconia, were immersed in the saliva for 2 h at 37 °C.

#### 2.5.2. Bacterial Culture

The antimicrobial activity of sterilized samples was tested against gram-positive *Streptococcus mutans* (*S. mutans*, UA159) bacteria and gram-negative *Porphyromonas gingivalis* (*P. gingivalis*, ATCC 33277) bacteria, which were provided by the Institute of Microorganisms, Chinese Academy of Sciences. The bacteria strains were maintained using brain–heart infusion (BHI) agar plate (Difco, MI, USA). The incubation condition for *S. mutans* was the standard cell condition (5% CO_2_, 95% humidified air, at 37 °C), and the incubation condition for *P. gingivalis* was the standard anaerobic condition (80% N_2_, 10% H_2_, 10% CO_2_, at 37 °C). After the exponential growth phase in the liquid medium, the bacterial cells were collected, centrifuged at 3000× *g* for 15 min, and washed two times with 0.1M PBS buffer. Bacterial suspensions with different final concentrations were shaken for 30 s (Vortex 2, IKA, Königswinter, Germany) to obtain single cells or pairs, then seeded onto the sample disks for further experiments. Each experiment was run in triplicate and repeated on three separate occasions.

#### 2.5.3. Spot-Assay Analysis

The antibacterial efficiency of silver-implanted zirconia was evaluated using a spot assay described by Suppi and Kasemets [37]. Briefly, the final concentration of bacteria suspensions was adjusted to 1 × 10^5^ CFU/mL before added onto saliva-coated zirconia disks. After 4 h of contact with disks, 10 μL of bacterial suspension was pipetted as a ‘spot’ onto the BHI agar plates to assess the viability of the bacteria cells. The agar plates were cultivated in standard cultivation conditions and the numbers of bacterial colonies were recorded.

#### 2.5.4. LIVE/DEAD Staining Assay

To evaluate the viability of the bacteria after seeding on silver-implanted zirconia, the LIVE/DEAD BacLight Bacterial Viability Kit (L-7012, Invitrogen, Carlsbad, CA, USA) was used. Briefly, staining components A (SYTO 9) and B (propidium iodide) were premixed and diluted with PBS at a ratio of 1.5:1.5:1000 *v*/*v*. The bacteria were seeded to the zirconia surface and cultivated for 24 h, then washed three times with PBS to remove non-adhered cells. A 300 μL mixed staining dilution was added to the zirconia surface. After the staining process, zirconia disks were observed using confocal laser scanning microscopy (CLSM; LSM710, Zeiss, Jena, Germany) at 100-fold magnification. The live cells were stained as green fluorescent and dead cells were stained as red fluorescent.

#### 2.5.5. MTT Colorimetric Assay

The MTT assay is based on the cleavage of MTT (3-(4,5-Dimethylthiazol-2-yl)-2,5-diphenyltetrazolium bromide) into a blue formazan by living cell enzymes. As previously described [38], the amount of formazan formed is positively correlated to the total viable cell counts. To prepare the MTT solution, 5 mg/mL MTT (Biosynth, Itasca, IL, USA) was dissolved in PBS and purified. The bacterial suspension with a final concentration of 1 × 10^8^ CFU/mL was added to saliva-coated zirconia disks and cultivated for 24 h. After removing the culture medium, the disks were washed three times with sterile PBS to remove non-attached bacterial cells. To perform the assay, 5 μL of MTT solution was added into 500 μL BHI broth culture medium. The mixed solution was added to the disks and incubated 3 h in the dark in standard cultivation conditions. After discarding the MTT solution, 500 μL DMSO (ThermoFisher Scientific, Waltham, MA, USA) was added to dissolve formazan crystals formed by viable cells. The optical density of the solution was determined at 570 nm using a microplate reader (ELX808, BioTek, Winooski, VT, USA). Solutions without seeding bacteria were used as blank controls. 

#### 2.5.6. Crystal Violet Assay

The total amount of biofilm formed on the zirconia disks was evaluated by using crystal violet (CV) assay. The bacterial suspension with a final concentration of 1 × 10^8^ CFU/mL was added to saliva-coated zirconia disks and cultivated for 48 h to form a biofilm. After discarding the growth medium, the samples were washed 3 times using PBS and fixed for 20 min at 37 °C using 2.5% glutaraldehyde. The crystal violet solution (Sigma-Aldrich, St. Louis, MO, USA) was used to stain the fixed biofilm by 10 min incubation at room temperature. The unbound dye was washed with gently running deionized water, and the bound dye was extracted using absolute ethanol. The amount of biofilm was measured at an optical density of 570 nm using a microplate reader. The background staining was corrected by subtracting the mean value for CV bound to negative controls.

### 2.6. Cellular Response

#### 2.6.1. Cell Culture

Primary HGFs were grown from tissue explants. Briefly, healthy gingival tissue obtained from patients who underwent periodontal surgery (crown-lengthening surgery) was cut into pieces (~1 mm^3^) and placed in 35 mm cell-culture dishes containing Dulbecco’s modified Eagle’s medium (DMEM; Gibco, Gaithersburg, MD, USA) supplemented with 10% fetal bovine serum (FBS; Gibco, Gaithersburg, MD, USA). Fibroblasts were obtained by trypsinization of the primary outgrowth of cells and were maintained and routinely passaged in 10 cm dishes in DMEM with 10% FBS and 1% antibiotic–antimycotic solution at 37 °C under a humidified atmosphere of 5% CO_2_ in 95% air. Cells in the third to sixth passage were used for the experiments.

#### 2.6.2. CCK-8 Assay

The cell proliferation on sample disks was evaluated by a quantitative colorimetric cell-counting kit-8 assay (CCK-8; Dojindo, Kyushu, Japan). Briefly, the HGF cells were seeded onto the disks at a density of 10^5^ cells. After incubation for 1 day, 3 days, and 7 days, the zirconia disks were washed with PBS three times. The CCK-8 diluted with cell-culture medium (1:10 ratio, *v*/*v*) was added to each well, followed by a 2 h incubation time at 37 °C. The supernatant was then transferred to 96-well plates and subjected to the optical density test using a microplate reader at 450 nm wavelength.

### 2.7. Statistical Analysis

The experimental data were obtained by three repeated experiments performed in triplicate. One-way analysis of variance (ANOVA) was used for comparisons among different groups. The level of significance was set at 0.05. The confidence level was set as 95%. Data analyses were performed using SPSS statistics software (ver. 25.0; SPSS Inc., Chicago, IL, USA).

## 3. Results

### 3.1. Surface Characteristics

#### 3.1.1. Surface Chemical Composition

**Figure 1:**
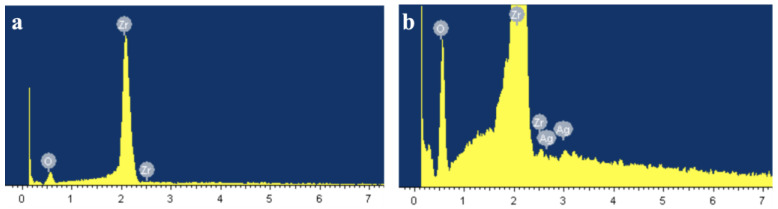
EDS results of (**a**) untreated surface and (**b**) silver-implanted surface at a nominal dose of 1 × 10^16^ ions/cm^2^.

The EDS results of the untreated zirconia and the silver-implanted zirconia at a nominal dose of 1 × 10^16^ ions/cm^2^ show different chemical elements on the surface. As shown in Figure 1 and Table 1, the untreated zirconia surface was composed solely of oxygen and zirconium, while several peaks of silver can be observed on the silver-implanted zirconia surface. The nominal dose of 1 × 10^16^ ions/cm^2^ will lead to a 0.62% silver atomic percentage increase on the zirconia surface.

**Figure 2:**
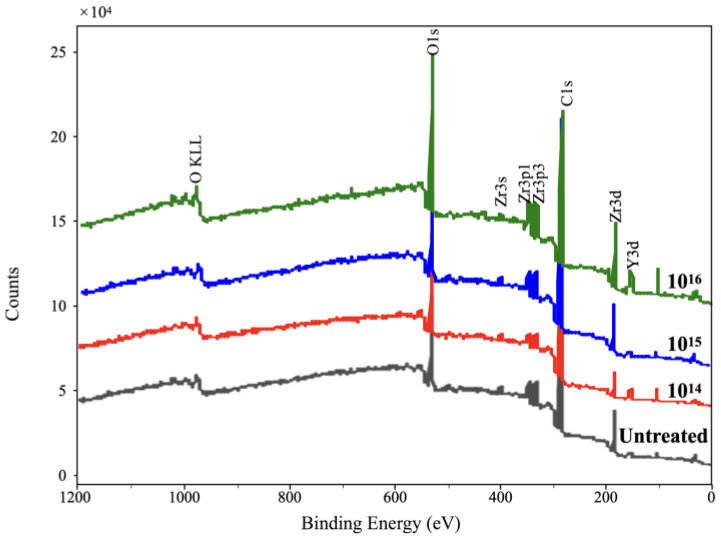
The XPS broad spectrum of zirconia disks.

**Figure 3:**
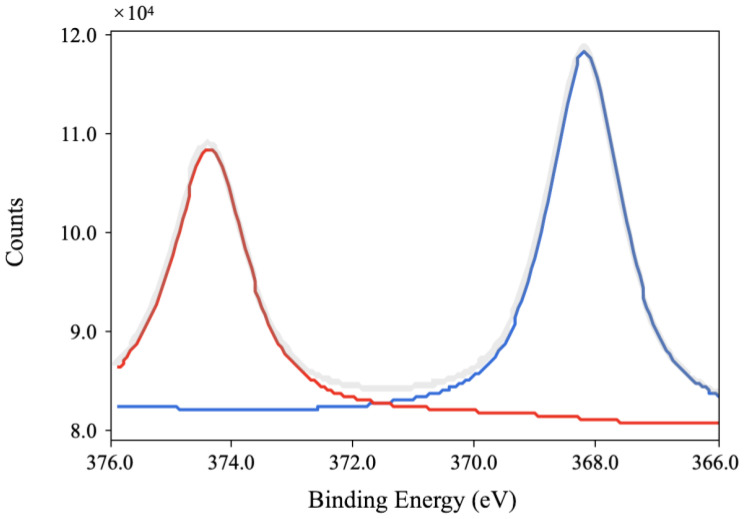
The Ag3d high-resolution image of silver-implanted zirconia disk (nominal dose of 1 × 10^16^ ions/cm^2^).

The XPS results (Figure 2) further reveal that surface composition changes after silver-ion implantation. Generally, the surface composition remained unchanged after the silver-ion implantation. However, as shown in Figure 3, a Ag3d spectrum can be observed on the high-resolution spectra of silver-implanted zirconia surface. The Ag3d doublet at 374.05 eV (Ag 3d_3/2_) and 368.04 eV (Ag 3d_5/2_) corresponds to metallic silver. These results confirm the successful implantation of silver nanoparticles to the zirconia surface.

#### 3.1.2. Surface Wettability

The results of surface wettability are shown in Table 2. Before silver implantation, the surface-water contact angle is 62.27 ± 2.52°, and the surface wettability remains after silver implantation. The silver-ion implantation does not change the overall surface wettability of zirconia disks. Since the surface free energy is related to the surface wettability [39], we presumed that surface free energy after the ion implantation remained as well.

#### 3.1.3. Silver-Ion Elution

For all liquid samples stored for 1, 3, 5, 7, 10, 14, 17, 21, 25, and 30 days, the silver-ion concentration is less than 0.1 ppb, which is barely detectable by ICP-MS. This result confirms that silver-implanted zirconia disks are stable, with little silver-ion elution to the surrounding environment.

### 3.2. Bacterial Response

#### 3.2.1. Bacterial Viability on the Silver-Implanted Zirconia Surface

**Figure 4:**
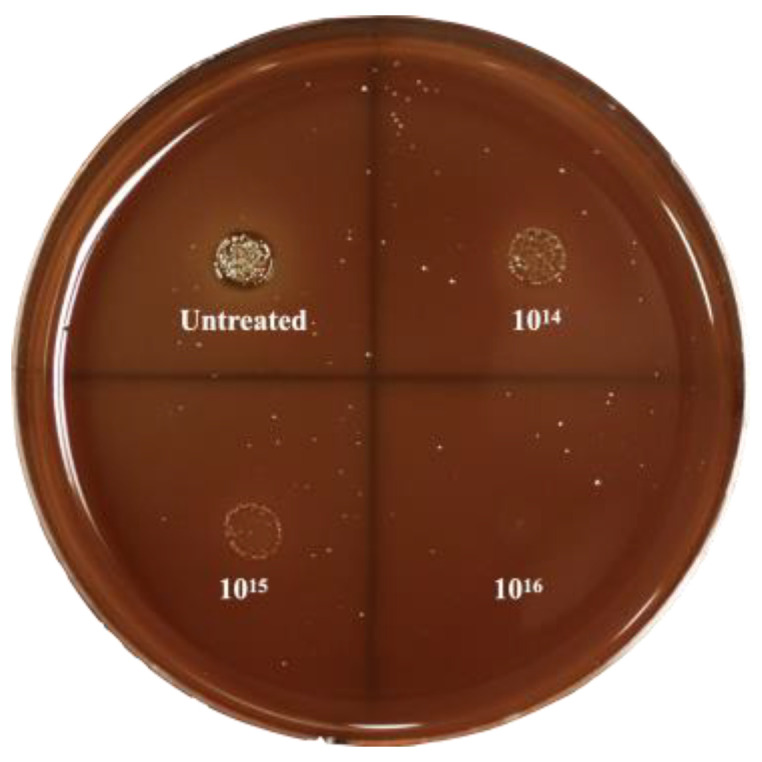
Spot-analysis result of *P. gingivalis*.

To first evaluate the bactericidal ability of silver-implanted zirconia against red-complex pathogen *P. gingivalis*, a spot analysis was performed. As shown in Figure 4, a significant decrease in bacterial load can be observed on the silver-implanted samples compared with the untreated disks. As the implant dose increases, there are fewer viable bacteria after coming into contact with the zirconia disks. This preliminary result indicates that silver-implanted zirconia shows a good bactericidal ability against gram-negative bacteria *P. gingivalis*.

**Figure 5:**
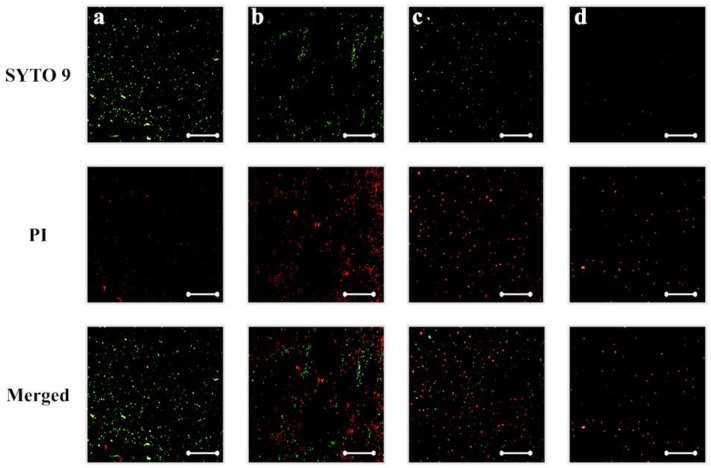
*P. gingivalis* viability on (**a**) untreated, (**b**) silver-implanted zirconia disks with a nominal dose of 10^14^, (**c**) 10^15^, and (**d**) 10^16^ ions/cm^2^, respectively. (Scale bar is 50 μm).

**Figure 6:**
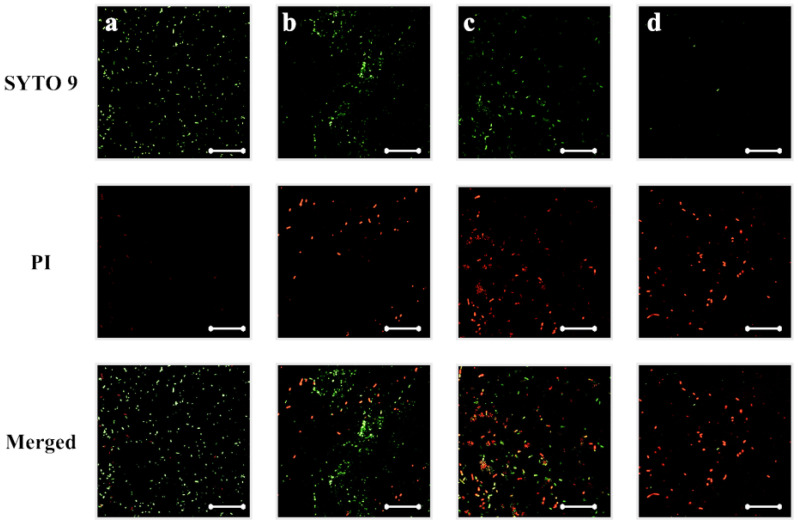
*S. mutans* viability on (**a**) untreated, (**b**) silver-implanted zirconia disks with a nominal dose of 10^14^, (**c**) 10^15^, and (**d**) 10^16^ ions/cm^2^, respectively. (Scale bar is 50 μm).

To further evaluate the bactericidal ability of silver-implanted zirconia, the LIVE/DEAD staining assay was performed. Figure 5 shows *P. gingivalis*’s viability on different zirconia surfaces. There are mainly viable (green fluorescent, stained by SYTO 9) bacterial cells on untreated zirconia surfaces. As the silver implant dose becomes higher, the number of viable bacteria cells decreases, and the number of inviable bacteria cells increases. The inviable bacterial cells were stained red by propidium iodide. For the gram-positive *S. mutans* bacteria, the same trend can be observed. As shown in Figure 6, the viability of bacterial cells decreases with the increase in silver implantation dose, which indicates an improved bactericidal effect with the increase in silver implantation dose. Taken together, an enhanced antibacterial effect can be observed with the accumulation of implanted silver nanoparticles on the zirconia surface.

#### 3.2.2. Bacterial Adhesion on Silver-Implanted Zirconia Surface

**Figure 7:**
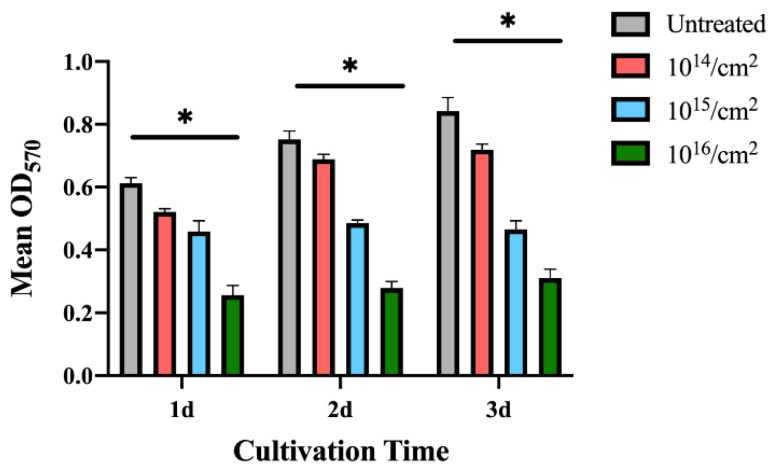
MTT results for *P. gingivalis.* (Data shown: mean and standard deviations; * indicates significant differences among different experimental groups, *p* < 0.05).

The MTT colorimetric assay was utilized to evaluate the bacterial adhesion to untreated zirconia disks or silver-implanted zirconia disks. As shown in Figure 7, the adhesion of *P. gingivalis* was inhibited on the silver-implanted zirconia surface after a one-day cultivation. The group of 10^16^/cm^2^ showed the least-adhered bacterial load on the surface among all groups. As the cultivation time extends, the same trend can be observed (fewer bacteria are adhering to the zirconia surface as the implantation dose increases).

**Figure 8:**
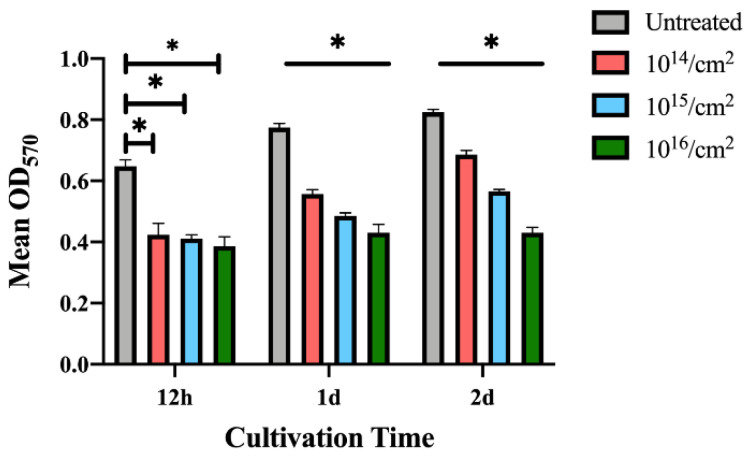
MTT results for *S. mutans.* (Data shown: mean and standard deviations; * indicates significant differences among different experimental groups, *p* < 0.05).

Since *S. mutans* grows faster than *P. gingivalis*, the MTT assays were performed 12 h after seeding to the sample surfaces. The results in Figure 8 show that *S. mutans*‘s adhesion was also interfered with on the silver-implanted zirconia surfaces. This adhesion-inhibition effect also shows a dose-dependent pattern, with fewer bacteria adhering to high implant-dose groups. Collectively, these results show that silver-implanted zirconia attenuates bacterial adhesion for both gram-positive and gram-negative pathogens. An enhanced inhibition can be seen as the implantation dose becomes higher.

#### 3.2.3. Bacterial-Biofilm Formation on the Silver-Implanted Zirconia Surface

**Figure 9:**
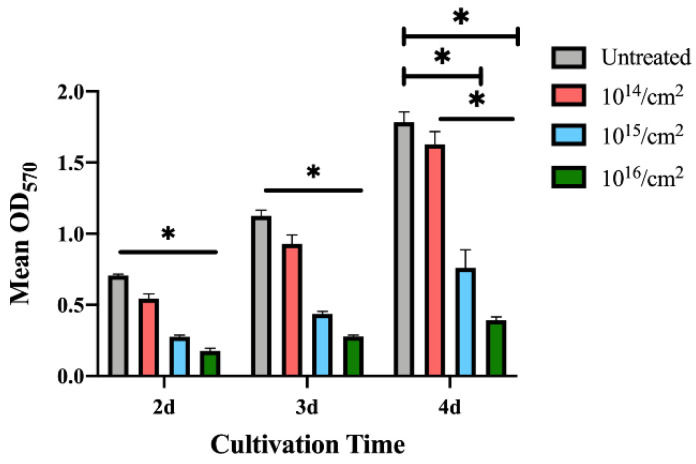
Biofilm formation on zirconia disks of *P. gingivalis.* (Data shown: mean and standard deviations; * indicates significant differences among different experimental groups, *p* < 0.05).

**Figure 10:**
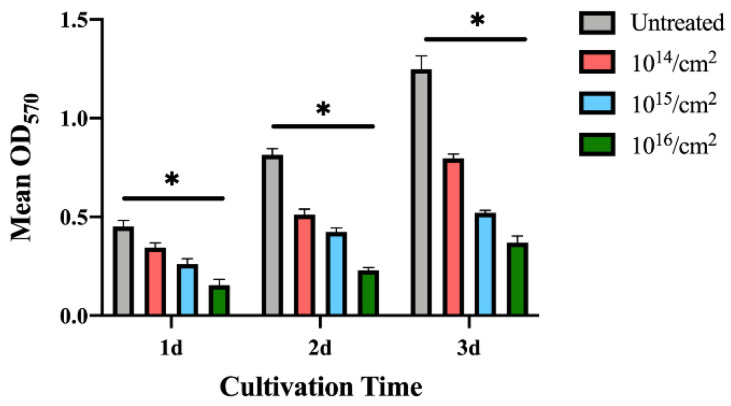
Biofilm formation on zirconia disks of *S. mutans.* (Data shown: mean and standard deviations; * indicates significant differences among different experimental groups, *p* < 0.05).

The biofilm formation on silver-implanted zirconia surfaces is shown in Figure 9 and Figure 10. For *P. gingivalis*, a total reduction in biofilm mass can be observed after a two-day cultivation. The zirconia disks with nominal doses of 10^15^/cm^2^ and 10^16^/cm^2^ accumulate fewer biofilms than the 10^14^/cm^2^ group. Indeed, after a four-day cultivation, there are no significant differences in the biofilms formed between the 10^14^/cm^2^ group and the untreated group. However, the 10^15^/cm^2^ and 10^16^/cm^2^ groups still show a biofilm-inhibition effect after a 4-day cultivation. This result indicates that, after a certain cultivation time, the group with an implant dose of 10^14^/cm^2^ might eventually lose its anti-biofilm formation ability.

The biofilm formed by *S. mutans* on the zirconia disks shows a more unified trend within 3 days of seeding bacteria to the sample surface. As shown in Figure 10, the 10^15^/cm^2^ and 10^16^/cm^2^ groups also show a good anti-biofilm-formation ability. The crystal violet results reveal that the decrease in biofilm formation can be observed on silver-implanted zirconia for both gram-positive and gram-negative bacteria.

### 3.3. Cellular Response by CCK-8 Assay

**Figure 11:**
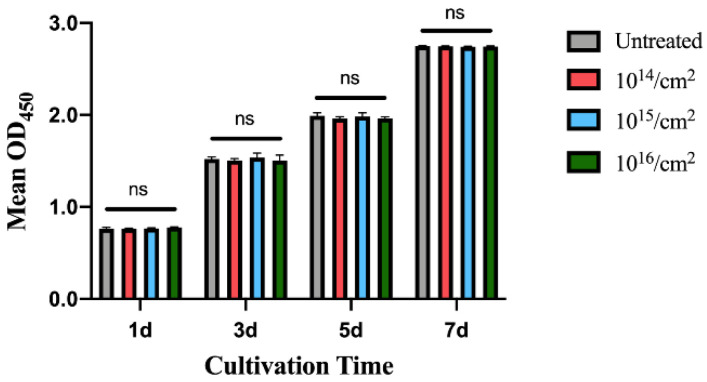
The CCK-8 results for human gingival fibroblast on zirconia disks. (Data shown: mean and standard deviations; ns: not significant).

To evaluate the cytotoxicity of silver-implanted zirconia, the CCK-8 colorimetric assay was performed. As shown in Figure 11, there is no significant difference between the untreated group and the silver-implanted groups. All groups show a similar growth rate, and this trend is retained after 7 days of incubation. The CCK-8 results show silver-implanted zirconia barely has any cytotoxicity to human gingival fibroblast, which is a crucial cell during soft-tissue wound healing after implant surgery and abutment installation.

## 4. Discussion

Implant-associated infection has been widely researched due to its high prevalence. Until now, most researchers and clinicians believed it was bacterially derived, thus lots of researchers have focused on the surface modification of implant materials to improve their antimicrobial efficacy. The surface modification of implant materials should show a great antimicrobial effect without releasing antibiotic agents at a fast rate, while being non-hazardous to the surrounding living human cells. Although many antimicrobial coatings have been presented, most of them are prone to degradation in the oral environment quickly. The method of silver nanoparticles embedded into zirconia by ion implantation is presented in this article. Using EDX, silver can be detected on the surface of zirconia. In the XPS results, the Ag3d doublet at 374.05 eV (Ag3d_3/2_) and 368.04 eV (Ag3d_5/2_) corresponds to metallic silver, indicating the nanoparticles are metallic silver. The ICP-MS test shows that silver nanoparticles remain stable and have little ion release (the leaching of silver ions is under the detection limit of 0.1 ppb even after 1 month of immersion). The low leaching property indicates silver ions will not accumulate in the tissues around implants; otherwise, it would result in cytotoxicity or other side effects.

This study investigates the antimicrobial ability of silver-nanoparticle-implanted zirconia, and the results show it can significantly enhance the antimicrobial ability in vitro and cause little harm to live human gingival fibroblasts. In antimicrobial assays, both the 1 × 10^15^ ions/cm^2^ and the 1 × 10^16^ ions/cm^2^ nominal-dose groups show positive anti-microbial properties on *P. gingivalis* and *S. mutans* in all assays, while the 1 × 10^16^ ions/cm^2^ group shows the strongest effects on bacterial inhibition. The LIVE/DEAD staining assay results demonstrated a decrease in live cells on the silver-implanted samples, and a decrease in the total number of cells was also observed. The crystal violet staining test shows the anti-biofilm activity of silver nanoparticles, which was in agreement with previously conducted studies [40,41]. The study did not choose the Zone of Inhibition (ZOI) assay for the reason that the antimicrobial effect of silver nanoparticles does not come from the leaching of silver ions from the material’s surface. It is believed that silver nanoparticles firmly attached to the zirconia surface can interact with microorganisms by direct contact. Furthermore, although the size of silver nanoparticles is important since it has an influence on cytotoxicity and antibacterial effects [20,42], it is hard to evaluate after the ion-implantation process, as the nanoparticles are embedded in the zirconia surface.

Although many research studies have investigated the antimicrobial activity of silver nanoparticles, the mechanism of the antibacterial activities and potential hazards remain unclear. However, possible theories have been made, as suggested by Marambio-Jones and Hoek et al. [43]: (a) uptake of free silver ions followed by disruption of ATP production and DNA replication, (b) reactive oxygen species (ROS) generation by silver nanoparticles and silver ions, and (c) direct damage to the cell membrane by silver nanoparticles. From the result of this study, it is believed that silver nanoparticles interact with microorganisms in the latter two ways, since silver ions are not released in a remarkable manner. The silver nanoparticles can produce high levels of ROS and free-radical species such as hydrogen peroxide, superoxide anion, hydroxyl radical, hypochlorous acid, and singlet oxygen [44,45]. The excessive ROS cannot be eliminated by antioxidant systems and will inhibit cell respiration and growth. Increased ROS levels will lead to an apoptosis-like response, which will cause DNA damage and, eventually, cell death. On the other hand, silver nanoparticles can anchor to the bacterial cell wall and, consequently, infiltrate it. This action will cause physical changes in the bacterial membrane, e.g., membrane damage, which can lead to cellular-content leakage and bacterial death [46,47].

Soft-tissue connection is crucial to implant dentistry. Ideal dental implant bio-materials require effective soft-tissue integration for long-term stability after implantation. A sound soft-tissue seal on the implant-abutment site can not only inhibit unwanted soft-tissue recession and marginal bone loss, but also resist bacterial invasion. Following implant surgery, HGFs are involved in the formation of connective tissues around the implant site. The CCK-8 proliferation assay indicates that silver nanoparticles show no obvious damage to HGFs’ proliferation. An effective HGF attachment to implant abutment plays a critical role in soft-tissue connection, and a strong attachment starts with a undisturbed initial proliferation. At the same time, the antimicrobial effect of silver-implanted surfaces can further enhance soft-tissue integration. The silver-implanted zirconia surface can provide an antibacterial effect without obvious cytotoxicity; however, as many cells are involved in the soft-tissue wound-healing process, more experiments should be carried out in the future to evaluate their response to the silver-implanted zirconia.

Taken together, the results suggest that nanostructured surfaces with silver nano-particles incorporated in zirconia may provide a better choice for dental implant abutments than traditional ones. Silver nanoparticles show a promising bactericidal ability that will be effective in the oral environment. However, only two pathogens were evaluated in this study. More pathogens, such as *Staphylococcus aureus* and other red-complex pathogens, should be studied further. Moreover, mono-culture in vitro studies are sometimes at odds with clinical reality. To avoid this, further in vivo studies to mimic clinical situations are required. Furthermore, the long-term release of silver ions and their safety for human beings need to be carefully evaluated. The release of silver ions in a different medium (such as saliva or blood serum) should also be evaluated in the future. The possible mechanical property changes (such as surface hardness or the Young’s modulus) after silver-ion implantation should also be investigated before its clinical usage.

## 5. Conclusions

In this work, we present an effective surface-modification technique for zirconia dental implant materials. Silver was implanted into zirconia with a nanostructured surface. Within the limitations of this in vitro study, the results suggest that silver-implanted zirconia has a strong antimicrobial effect against oral microorganisms, including *P. gingivalis* and *S. mutans*. It also shows no harm to human gingival fibroblast proliferation. Silver-implanted zirconia shows promise for both preventing peri-implant lesions and forming peri-implant soft-tissue attachments in clinical application.

## Figures and Tables

**Table 1:** Element percentage change on zirconia disks after silver-ion implantation.

Elements	Untreated Zirconia		Silver-Implanted Zirconia (1 × 10^16^ ions/cm^2^)	
	Weight Percentage (%)	Atomic Percentage (%)	Weight Percentage (%)	Atomic Percentage (%)
O	20.3	59.3	22.5	62.4
Zr	79.7	40.7	76.0	37.0
Ag	-	-	1.5	0.6

**Table 2:** Surface-water contact-angle results of zirconia disks (Mean ± SD).

Group	Untreated	1 × 10^14^/cm^2^	1 × 10^15^/cm^2^	1 × 10^16^/cm^2^
Surface-water contact angle (°)	62.27 ± 2.40	63.18 ± 2.47	60.51 ± 2.68	62.83 ± 3.73
Image	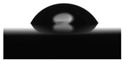	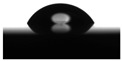	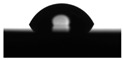	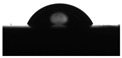

## Data Availability

Research data are available upon request to the authors.
